# Acquired resistance to EGFR tyrosine kinase inhibitors alters the metabolism of human head and neck squamous carcinoma cells and xenograft tumours

**DOI:** 10.1038/bjc.2015.86

**Published:** 2015-03-05

**Authors:** M Beloueche-Babari, C Box, V Arunan, H G Parkes, M Valenti, A De Haven Brandon, L E Jackson, S A Eccles, M O Leach

**Affiliations:** 1 Cancer Research UK Cancer Imaging Centre, Division of Radiotherapy and Imaging, The Institute of Cancer Research, London and The Royal Marsden NHS Foundation Trust, Sutton, Surrey SM2 5PT, UK; 2 Division of Cancer Therapeutics, The Institute of Cancer Research, London SM2 5NG, UK

**Keywords:** acquired drug resistance, biomarkers, EGFR tyrosine kinase inhibitors, head and neck cancer, metabolism

## Abstract

**Background:**

Acquired resistance to molecularly targeted therapeutics is a key challenge in personalised cancer medicine, highlighting the need for identifying the underlying mechanisms and early biomarkers of relapse, in order to guide subsequent patient management.

**Methods:**

Here we use human head and neck squamous cell carcinoma (HNSCC) models and nuclear magnetic resonance (NMR) spectroscopy to assess the metabolic changes that follow acquired resistance to EGFR tyrosine kinase inhibitors (TKIs), and which could serve as potential metabolic biomarkers of drug resistance.

**Results:**

Comparison of NMR metabolite profiles obtained from control (CAL^S^) and EGFR TKI-resistant (CAL^R^) cells grown as 2D monolayers, 3D spheroids or xenograft tumours in athymic mice revealed a number of differences between the sensitive and drug-resistant models. In particular, we observed elevated levels of glycerophosphocholine (GPC) in CAL^R^ relative to CAL^S^ monolayers, spheroids and tumours, independent of the growth rate or environment. In addition, there was an increase in alanine, aspartate and creatine+phosphocreatine in resistant spheroids and xenografts, and increased levels of lactate, branched-chain amino acids and a fall in phosphoethanolamine only in xenografts. The xenograft lactate build-up was associated with an increased expression of the glucose transporter GLUT-1, whereas the rise in GPC was attributed to inhibition of GPC phosphodiesterase. Reduced glycerophosphocholine (GPC) and phosphocholine were observed in a second HNSCC model probably indicative of a different drug resistance mechanism.

**Conclusions:**

Our studies reveal metabolic signatures associated not only with acquired EGFR TKI resistance but also growth pattern, microenvironment and contributing mechanisms in HNSCC models. These findings warrant further investigation as metabolic biomarkers of disease relapse in the clinic.

Epidermal growth factor receptor (EGFR/HER1) is over-expressed in 95% of head and neck squamous cell carcinomas (HNSCC) and high levels are correlated with poor patient prognosis and decreased overall survival (Grandis *et al*, 1998; Ang *et al*, 2002), suggesting that the disease is EGFR-driven and making a strong case for targeting EGFR in HNSCC. Disappointingly, however, clinical evidence to date shows that EGFR-targeted agents, including monoclonal antibodies and tyrosine kinase inhibitors (TKIs), have little efficacy in HNSCC as a monotherapy, raising the possibility that alternative survival pathways may be activated in this disease (Box *et al*, 2013a, 2013b). To address this, new strategies for targeting EGFR in HNSCC are being evaluated clinically including the use of pan-HER inhibitors and rational drug combinations that target several signalling pathways simultaneously (Agulnik, 2012).

A key challenge for TKIs (including those targeting EGFR) in cancer is the development of acquired resistance, with many patients showing disease progression not long after the initial response (Wagle *et al*, 2011; Lovly and Shaw, 2014). Thus, the elucidation of underlying molecular mechanisms, as well as early biomarkers of patient relapse, is key for identifying subsequent therapies that may delay or indeed prevent further disease progression, and ultimately improve long-term survival.

Studies have shown that treatment failure is, in many cases, due to cancer cells acquiring new genetic and molecular alterations that enable them to survive in the face of therapeutic stress (Holohan *et al*, 2013). Additionally, transformed tumour cell metabolism may be equally important in this regard since alterations in key processes such as glucose, glutamine, nucleotide, fatty acid and phospholipid metabolism allow cancer cells to adapt to their growth demands and thrive in a hostile microenvironment (Bailey *et al*, 2012; Ward and Thompson, 2012). Several oncoproteins and oncogenic signalling pathways directly regulate vital components of the metabolic network, thus providing a link between the molecular abnormalities of cancer and the transformed metabolic phenotype (Deberardinis *et al*, 2008; Vander Heiden *et al*, 2009; Vousden and Ryan, 2009).

Altered metabolism is a hallmark of cancer (Hanahan and Weinberg, 2011) and metabolic features of cancer are being explored for diagnosis and staging as well as for informing on disease response and resistance to therapy (Beloueche-Babari *et al*, 2010; Beloueche-Babari *et al*, 2011). In this context, metabolic imaging and/or profiling techniques such as positron emission tomography, nuclear magnetic resonance (NMR) spectroscopy and mass spectrometry play an important role in translating findings from pre-clinical studies to humans.

We have developed a model of acquired resistance to EGFR TKIs based on the human HNSCC cell line CAL 27, and shown that a resistant derivative (CAL^R^) exhibits a distinct protein expression signature compared with its sensitive counterpart (CAL^S^) (Box *et al*, 2013a). Because of the central role that metabolism plays in tumour biology (Hanahan and Weinberg, 2011), we explore here the metabolic features of EGFR TKI sensitivity in CAL^S^ and CAL^R^ cells grown as 2D monolayers, 3D spheroids and tumour xenografts by using NMR spectroscopy. Our aim is to assess the value of 3D cultures, compared with 2D or xenograft tumour models, for the discovery of potential metabolic signatures underlying resistance that could potentially be developed as minimally invasive metabolic biomarkers of EGFR TKI resistance, and to interrogate the influence of the microenvironment on metabolic readouts.

## Materials and methods

### Cell culture and *in vitro* experiments

CAL^S^/CAL^R^ and PJ^S^/PJ^R^ HNSCC cell lines were generated and maintained as previously described (Box *et al*, 2013a). CAL^R^ and PJ^R^ cells are stably resistant to EGFR TKIs and were thus routinely grown in gefitinib-free media. Cells were screened regularly for mycoplasma and cultured for no longer than ten passages. 2D cell counts and diameter measurements were performed on a Beckman Coulter Vi-Cell Cell Viability Analyser.

3D CAL^S^ and CAL^R^ tumour spheroids were generated by plating 4000 cells per well in normal growth medium in round bottom ultra-low attachment 96-well plates (Corning) as previously documented (Vinci *et al*, 2013). After 4 days, spheroids were fed by 50% medium replenishment and harvested for analysis after a further 3 days. Spheroid diameters were measured on a Celigo cytometer (Nexcelom Bioscience). Gefitinib was purchased from ChemieTek.

### Human tumour xenograft models

CAL^S^ and CAL^R^ subcutaneous tumours were established as described previously (Box *et al*, 2013a). Tumours were measured across two perpendicular diameters using callipers, and volumes were obtained from the following formula: *V*=4/3*π* [(*d*1+*d*2)/4]^3^. Once an appropriate tumour volume (∼350 mm^3^) was attained (∼2 weeks for CAL^R^ and ∼4 weeks for CAL^S^), tumours were excised, carefully dissected clear of surrounding skin and fat, snap-frozen in liquid nitrogen then stored at −80 °C until further processing for western blotting or *in vitro* NMR spectroscopy. All experiments were performed in accordance with UK Home Office regulations under the Animals (Scientific Procedures) Act 1986 and UK National Cancer Research Institute (NCRI) Guidelines for the Welfare and Use of Animals in Cancer Research (Workman *et al*, 2010).

### Metabolic evaluation of cells and tumours

For metabolic analysis by NMR spectroscopy, 2D subconfluent cell monolayers (∼5 × 10^7^ cells) were extracted using a dual phase method in equal volumes of cold methanol, chloroform and water as previously described (Beloueche-Babari *et al*, 2012). For the 3D experiments, individual 7-day old spheroids (from six 96-well ULA plates/cell line) were pooled and extracted using a similar approach. Xenograft tumour material (∼150 mg) was ground under liquid nitrogen in a pestle and mortar then a dual phase extraction was used, as described above. After phase separation, the aqueous fraction from each sample was lyophilised and reconstituted in 540 *μ*l of D_2_O containing 3-(trimethylsilyl) propionic-2,2,3,3-d_4_ acid (TSP) as an internal ^1^H NMR quantitation and chemical shift reference. For ^31^P NMR measurements, 60 *μ*l of a D_2_O solution containing EDTA and methylenediphosphonic acid (MDPA, internal standard) to a final concentration of 10 and 0.43 mM respectively (pH 8.2) was added.

### NMR spectroscopy data acquisition and processing

^1^H and ^31^P NMR spectroscopy data were acquired on a Bruker 500 MHz spectrometer at 25 °C. Acquisition and processing of spectra were performed as previously described (Beloueche-Babari *et al*, 2012). Metabolite content was measured by peak integration relative to TSP (^1^H NMR) or MDPA (^31^P NMR) and metabolite content normalised to cell number and volume for 2D cultures, and wet tissue weight for tumour samples. Spheroid data were expressed as ratios of metabolites.

### Multivariate analysis of NMR spectroscopy data

^1^H NMR data from CAL^R^ and CAL^S^ tumours were subjected to unbiased metabolic profiling using principal component analysis (PCA), a method that uses the original variables (metabolite peaks) to derive a new smaller set of orthogonal (uncorrelated) variables, or principal components, that explain the variance in the original data set. For this, spectra were phased and baseline corrected then integrated in spectral regions (bins) of 0.04 p.p.m. within the 0.8–4.38 p.p.m. excluding the residual methanol peak at 3.36 ppm. Integrals from individual spectral bins were normalised to the sum of total integrals obtained and processed in SIMCA v13.0 (Umetrics – Umeå, Sweden) using a PCA model.

### Western blotting

Cells were lysed as described previously (Rogers *et al*, 2009) except that tumour spheroids were stored at −80 °C overnight (to aid lysis) prior to centrifugation. Tumour samples were processed as previously documented (Beloueche-Babari *et al*, 2013). For analysis of GLUT-1 expression, proteins were electrophoresed under non-reducing conditions then transferred to PVDF membranes. Blots were probed with antibodies to GLUT-1 (Merck Millipore) or *β*-actin as a loading control (Abcam).

### Glycerophosphocholine-phosphodiesterase (GPC-pd) assay

GPC-pd enzymatic activity was monitored by ^1^H NMR by using a modification of a previously described method (Iorio *et al*, 2010). 3 × 10^7^ cells were pelleted, washed and homogenised then sonicated at 4 °C in 0.5 ml 100 mM Tris-HCl (pH 7.2) buffer containing 10 mM DTT and 1 mM EDTA in D_2_O. Following a 30 min centrifugation at 12 000 r.p.m., supernatants containing the cytosolic fractions were transferred to a 5 mm NMR tube. GPC-pd was assayed at 30 °C by monitoring the ^1^H NMR-observed build up of choline over time (35 min) following the addition of exogenous GPC and MgCl_2_ at a final concentration of 1.2 mM and 10 mM, respectively.

### Statistical analysis

For comparison of metabolite levels, Student's *t*-test was used with *P*-values of ⩽0.05 considered to be statistically significant. Data represent the mean±s.e.

## Results

### Acquired resistance to EGFR TKI in HNSCC alters tumour metabolism

We have previously reported that CAL^R^ cells exhibit a faster growth rate compared with CAL^S^ when grown as subcutaneous xenograft tumours but not when cultured as monolayers *in vitro* (Box *et al*, 2013a). Interestingly, when grown as 3D spheroids, CAL^R^ grew more rapidly than CAL^S^ cells with average spheroid volumes at day 7 of 31±3 and 17±4 × 10^6^ *μ*m^3^ (*n*=5 biological repeats, *P*=0.02), respectively, indicative of a more aggressive growth phenotype as observed *in vivo*.

We hypothesised that our cell line models with distinct growth properties would possess different metabolic characteristics. Thus, prior to assessing metabolic differences associated with the resistance phenotype we explored the extent of any baseline metabolic variations between the three growth conditions: 2D, 3D and xenograft tumours, within each cell line.

**Figure 1:**
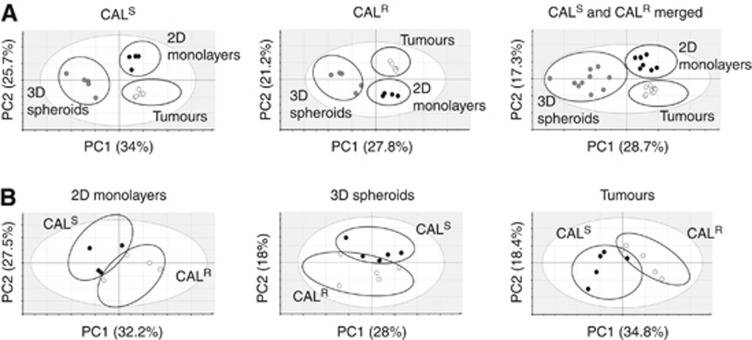
**Unbiased metabolomic profiling of CAL^S^ and CAL^R^ tumour models.** (**A**) 2D PCA score scatter plots showing a separate clustering for ^1^H NMR data from cells grown as 2D monolayers, 3D spheroids and xenograft tumours within the CAL^S^ and CAL^R^ cell lines separately and when the data are merged. (**B**) 2D PCA score scatter plots showing separate clustering for CAL^S^ and CAL^R 1^H NMR data points within the 2D cell model, 3D spheroids and tumours. PC1 and PC2 are the two most important principal components explaining the variation in the data (shown as percentages in the *x* and *y* axes).

As shown in Figure 1A, unbiased multivariate analysis with PCA of the ^1^H NMR spectral data indicated that the 2D cells, 3D spheroids and tumours exhibit separate clustering within each cell line, consistent with a distinct metabolic phenotype. The clustering was maintained even when data from CAL^S^ and CAL^R^ were merged, suggesting strong model-dependent patterns. The score scatter plots indicate that the variation along the PC1 axis is driven by differences between the 2D and tumour data *vs* the spheroid data while the variation along the PC2 axis is driven by differences between the 2D *vs* tumour data with spheroid data overlapping between the two. Thus, despite arising from the same cells of origin, the three experimental models used in this study have unique metabolic features which are likely to be a reflection of their growth phenotype and microenvironment.

The metabolic characteristics of acquired EGFR TKI resistance were assessed with PCA of the ^1^H NMR data derived from CAL^S^ and CAL^R^ cells within each model. The separate clustering of the data points corresponding to CAL^S^ and CAL^R^ on the score scatter plots in Figure 1B indicates a distinct metabolic profile for the sensitive and the EGFR TKI-resistant cells in every model.

**Figure 2:**
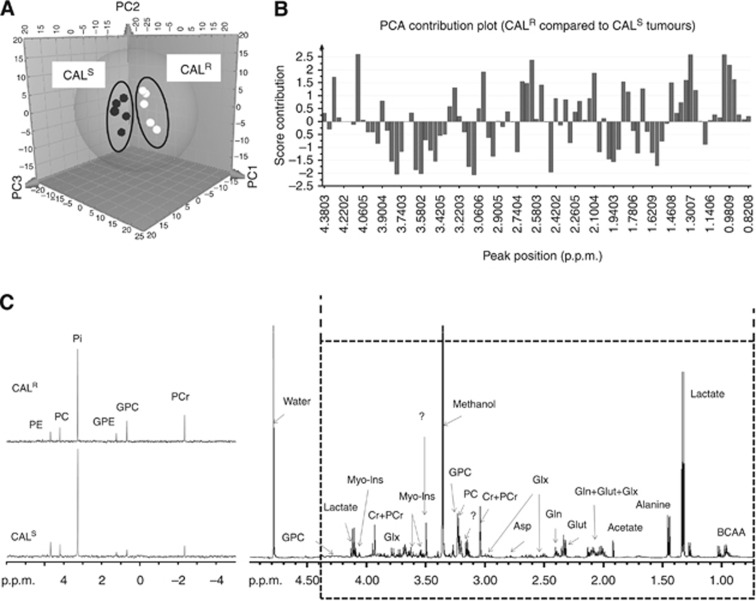
**NMR profiling of CAL^S^ and CAL^R^ tumours.** (**A**) Three-dimensional PCA score scatter plot showing separate clustering for ^1^H NMR data from CAL^S^ and CAL^R^. (**B**) Score contribution plot showing changes in the ^1^H NMR peaks (and related metabolites) accounting for the differences between CAL^R^ and CAL^S^ tumours (plot obtained using the group-to-group comparison option in SIMCA). Positive scores represent increased levels, while negative scores indicate decreased levels in CAL^R^ relative to CAL^S^. (**C**) Representative ^31^P NMR spectra showing the differences in ^31^P-containing metabolites between CAL^S^ and CAL^R^ tumours. Abbreviations: Asp=aspartate; BCAA=branched-chain amino acids; Cr=creatine; PCr=phosphocreatine; PC=phosphocholine; PE=phosphoethanolamine; GPC=glycerophosphocholine; GPE=glycerophosphoethanolamine; Pi=inorganic phosphate; Gln=glutamine; Glut=glutamate; Glx=glutathione; Myo-Ins=myo-inositol; ?=unidentified peak.

The clearest separation was obtained in the tumours which showed that variability in the data could be described according to three main principal components, PC1, PC2 and PC3 (Figure 1B and 2A), that between them explain ∼68% of the total variance (PC1: 34.8%, PC2: 18.4%, PC3: 15.1%). The resonances that appeared to be key in the separation between the CAL^S^ and CAL^R^ profiles include lactate, branched-chain amino acids (BCAAs), choline metabolites, acetate, myo-inositol, glutamine/glutamate and creatine (Cr)+phosphocreatine (PCr), as shown in Figure 2B.

To validate the metabolite changes identified in the PCA, we performed a targeted analysis of the data by integrating the individual peaks in the ^1^H NMR spectra. As shown in Table 1, and in agreement with the PCA method, univariate ^1^H NMR revealed a number of metabolic alterations in CAL^R^ xenograft tumours compared with their CAL^S^ counterpart. Specifically, the levels of GPC, lactate, BCAAs, alanine and aspartate were significantly elevated in CAL^R^ relative to CAL^S^ tumours. Total choline, which is predominantly comprised of GPC, phosphocholine (PC) and free choline, was also increased in CAL^R^ compared with CAL^S^. The levels of Cr/PCr, acetate and glutamate showed a trend towards an increase, while myo-inositol showed a trend towards a decrease in CAL^R^ compared with CAL^S^ tumours but these effects did not reach statistical significance.

^31^P NMR analysis of CAL^S^ and CAL^R^ tumour extracts corroborated the elevation in GPC and PCr levels and additionally revealed a rise in glycerophosphoethanolamine (GPE) and a fall in phosphoethanolamine (PE) levels in CAL^R^ relative to CAL^S^ tumours (Table 1 and Figure 2C). NTP levels were not reproducibly detectable in the ^31^P NMR spectra of tumour extracts, but the consistent and significant increase in CAL^R^ PCr levels suggests that the drug-resistant tumours have improved bioenergetics.

### Relationship between metabolism, drug resistance, growth environment and phenotype

To investigate the significance of the metabolic changes observed in relation to the acquired drug resistance phenotype and how this is impacted on by the microenvironment and the differential growth rates of the tumours, we assessed whether the metabolic signature of drug resistance identified in the xenograft tumours could also be present in cells grown *in vitro*. For these experiments we compared cells cultured as (a) 2D monolayers with comparable CAL^S^/CAL^R^ growth rates and (b) 3D spheroids with a faster CAL^R^ growth rate, reminiscent of the *in vivo* xenograft model.

Univariate ^1^H NMR spectroscopy analysis of 2D cell extracts showed that, consistent with the tumour data, CAL^R^ cells exhibited higher levels of GPC relative to CAL^S^ cells while BCAAs lactate, Cr+PCr, aspartate and alanine, which were increased in the CAL^R^ tumours, were not significantly changed in the 2D cell cultures (Table 2).

For the analysis of 3D spheroids, as it was not possible to obtain an accurate estimate of cell number, we normalised the metabolite content relative to the PC signal since this was found to be comparable in the tumours as well as the 2D cultured cells.

The data showed that, similar to the tumour profiles, the ratios of GPC/PC, PCr+Cr/PC, alanine/PC and aspartate/PC were significantly elevated. However, in contrast to the observations made in solid tumours, the ratio of BCAA/PC appeared to decrease in CAL^R^ compared with CAL^S^ spheroids, although this was not statistically significant, while lactate/PC remained constant within experimental uncertainly (Table 2).

**Figure 3:**
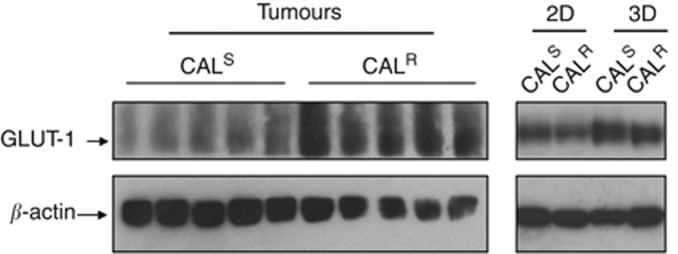
**GLUT-1 expression in CAL^S^ and CAL^R^ tumour models.** Western blots showing an elevated GLUT-1 protein expression in CAL^R^ relative to CAL^S^ xenograft tumours but not in 2D monolayers or 3D spheroids. The individual lanes in the tumour blot represent samples from individual xenografts.

The increased lactate observed in tumours but not 2D or 3D *in vitro* cultures could suggest a shift towards glycolytic metabolism (also known as the Warburg effect) *in vivo*. To investigate this possibility we assessed the protein expression levels of the glucose transporter GLUT-1. Our data indicate that CAL^R^ have upregulated GLUT-1 expression compared with CAL^S^ tumours, consistent with an increased Warburg effect. Interestingly, this difference was not observed in cells grown as 2D monolayers or 3D spheroids (Figure 3), where lactate levels were unchanged.

**Figure 4:**
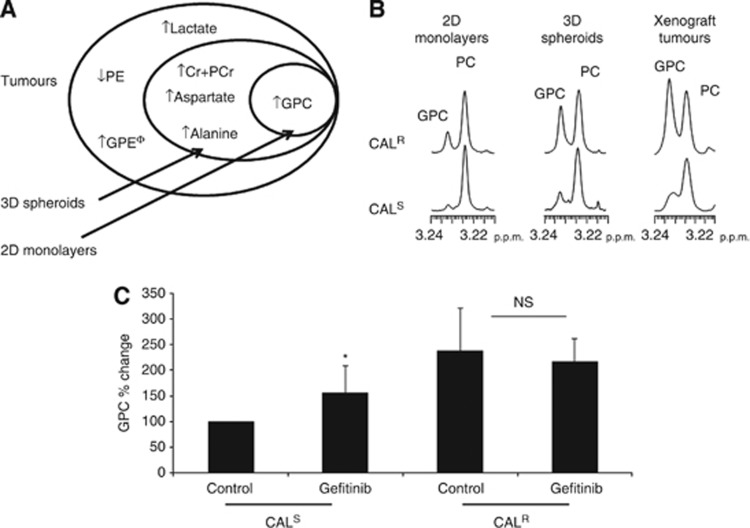
**Comparison of metabolite changes induced in EGFR TKI resistance models.** (**A**) Diagram summarising the metabolite signature observed in CAL^R^ relative to CAL^S^ tumours and its presence in 2D and 3D *in vitro* cultures. (**B**) ^1^H NMR spectra of the choline region showing an increased GPC in CAL^R^ relative to CAL^S^ models. (**C**) Changes in GPC following an acute exposure to gefitinib (24 h, 1 *μ*M) in CAL^S^ and CAL^R^ cell lines grown in 2D. ^Φ^: not measured in spheroids or 2D monolayers, *P=0.04, NS: *P*=0.58, *n*=3 at least.

Comparison of the data obtained from 2D cultures, 3D spheroids and xenograft tumours indicate that, relative to CAL^S^, CAL^R^ cells displayed increased levels of lactate, BCAA and decreased PE *in vivo* only, elevated PCr, alanine and aspartate levels in 3D spheroids and tumours, and increased GPC in all three experimental models (Figure 4A and B). Thus the rises in lactate and GLUT-1 expression, together with increased BCAA and decreased PE are associated with the *in vivo* growth environment while the increase in alanine, aspartate and Cr+PCr is associated with the aggressive growth phenotype seen in 3D and *in vivo*. The build up of GPC is associated with EGFR TKI resistance independently of differences in growth rates or environment.

To assess the dynamics of the change in GPC, we monitored the levels of this metabolite following acute exposure to drug. ^1^H NMR spectroscopy of cells treated with 1*μ*M gefitinib (EGFR TKI) for 24 h showed that GPC levels increased in CAL^S^ (to 157±21% relative to controls, *P*=0.04) but not in CAL^R^ cells (95±13% relative to controls, *P*=0.36), which were unresponsive to drug as determined by the effects of treatment on cell counts: 94±5% of controls (*P*=0.25) in CAL^R^ compared with 70±5% (*P*=0.001) in CAL^S^ (Figure 4C). Thus, the rise in GPC appears to be an early metabolic consequence of exposure to gefitinib, which is sustained with chronic exposure when cells acquire long-term drug resistance.

### EGFR TKI resistance in the CAL^S^/CAL^R^ model is associated with inhibition of GPC-pd

GPC is produced from the breakdown of the membrane phospholipid phosphatidylcholine (PtdCho) in a 2-step reaction mediated via phospholipase A and lysophospholipase, which concomitantly leads to the formation of free fatty acids (FFAs). GPC is further metabolised to glycerol-3 phosphate and choline in a reaction catalysed by GPC phosphodiesterase (GPC-pd).

To determine the metabolic processes underpinning the elevation in GPC levels, we initially assessed the levels of FFA signals in the ^1^H NMR spectra of the organic phase of cells extracts. No significant differences were recorded between the profiles of CAL^S^ and CAL^R^ cells indicating that the rates of PtdCho breakdown were comparable in the two cell lines (Supplementary Table S1).

**Figure 5:**
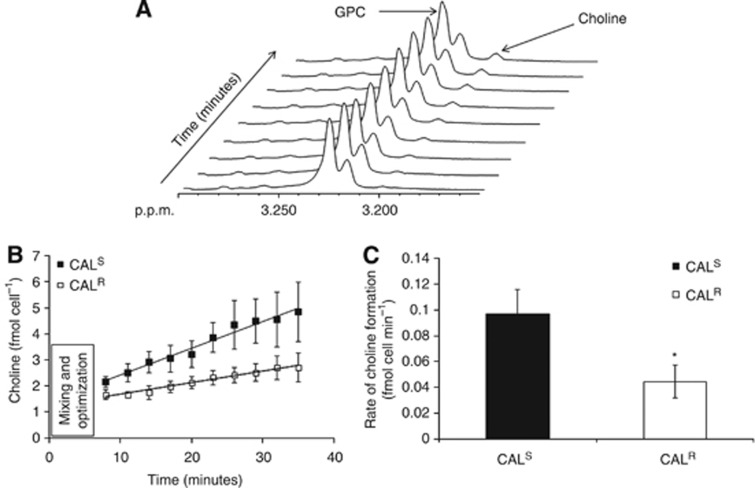
**GPC-pd activity assay in CAL^R^ and CAL^S^ monolayers.** (**A**) Sequential ^1^H NMR spectra from the GPC-pd assay showing the changes in choline and GPC resonances over time. (**B**) Build up of choline in 2D CAL^R^ and CAL^S^ cells over time (total of 35 min) following addition of GPC. (**C**) Quantification of GPC-pd activity shows a decrease in CAL^R^ relative to CAL^S^ cells. *P=0.03, *n*=4.

The breakdown of GPC via GPC-pd was next investigated using a ^1^H NMR-based assay that monitored the formation of choline over time as a measure of GPC-pd activity (Figure 5A and B). This showed that the rate of choline formation as a result of GPC hydrolysis was 97±19 pmol per cell per min in CAL^S^ monolayers, decreasing to 45±13 pmol per cell per min in CAL^R^ monolayers (*P*=0.03, *n*=4), consistent with lower GPC-pd activity in this cell line (Figure 5C). Taken together these findings show that the increase in GPC content in the EGFR TKI-resistant CAL^R^ cells is likely to be due to a reduction of its metabolism by GPC-pd.

### The metabolic signatures of acquired EGFR TKI resistance may be mechanism-dependent

Finally, to test the generalisability of our observations we assessed a second sensitive/resistant HNSCC model that we have previously described: the PJ^S^ and PJ^R^ cell lines (Box *et al*, 2013a). DNA staining and flow cytometry showed polyploidy in PJ^R^ cells, a phenomenon that was not observed in PJ^S^, CAL^S^ or CAL^R^ cell lines (supplementary Figure S1). Polyploidy has previously been linked to drug resistance (Puig *et al*, 2008; Shen *et al*, 2008), suggesting a potential difference in the underlying mechanisms of acquired resistance to EGFR TKIs between PJ^R^ and CAL^R^ models. Our ^1^H NMR data show that, unlike the case for CAL^R^ *vs* CAL^S^, drug-resistant PJ^R^ cells in standard 2D cultures exhibited reduced levels of GPC and PC compared with the sensitive PJ^S^ cells with no significant changes in the levels of lactate, alanine, glutamate or BCAA (Table 3). Thus, acquired resistance to EGFR TKIs in the PJ^R^/PJ^S^ model is associated with a differential metabolic response compared with the CAL^R^/CAL^S^ model, which is likely to be related to the differences in accompanying cellular processes and potentially the underlying drug resistance mechanisms.

## Discussion

Acquired resistance to TKIs is a major challenge in personalised cancer medicine and the identification of mechanisms, as well as early biomarkers of patient relapse, will enable a timely switch to alternative therapies prior to further disease progression.

In this study we used TKI sensitive (CAL^S^) and resistant (CAL^R^) human HNSCC cells grown as standard 2D monolayers, 3D spheroids or tumour xenografts alongside NMR spectroscopy to explore metabolic characteristics of acquired resistance to EGFR TKIs which, if independently validated, could have potential as minimally invasive biomarkers, and to assess the influence of the microenvironment on the metabolic readouts.

^1^H and ^31^P NMR spectroscopy combined with PCA and univariate metabolite analysis of tumour extracts revealed a number of metabolic differences between CAL^R^ and CAL^S^ tumours, in particular increased lactate, GPC, BCAA, alanine, aspartate, PCr and decreased PE. These changes are likely to reflect differences in metabolism between CAL^R^ and CAL^S^ tumour cells since the extent of stromal enrichment, as revealed by haematoxylin and eosin staining (Supplementary Figure S2), was comparable between the CAL^S^ and CAL^R^ tumours, indicating that any contributions from non-tumour cells to the metabolic readouts should be similar between the two models.

Assessment of this metabolic signature in *in vitro* models indicated that in 3D spheroids, where CAL^R^ had a more aggressive growth phenotype relative to CAL^S^, increased GPC, Cr/PCr, aspartate as well as alanine were also observed alongside drug resistance. On the other hand, in 2D cultures, where CAL^R^ did not exhibit a faster growth rate than CAL^S^ cells, only an increase in GPC was detectable. Taken together, these data suggest that the increase in tumour lactate and BCAA and decreased PE, only detectable *in vivo*, could reflect contribution from the tumour microenvironment, while the increase in PCr, aspartate and alanine levels observed in the CAL^R^ tumours and 3D spheroids may be linked to the aggressive growth phenotype they exhibit in comparison with their CAL^S^ counterpart. In contrast, the rise in GPC, which was observed in all three growth conditions irrespective of their growth rate, is likely to be associated with acquired resistance to EGFR TKI in our HNSCC model. Our data also show that the metabolic phenotype exhibited by CAL^R^ and CAL^S^ xenograft tumours was reflected more accurately by the 3D spheroids than by the 2D monolayers, which reinforces the increased value of 3D cultures as biologically relevant experimental models of cancer (Vinci *et al*, 2012).

Increased glycolytic metabolism has been reported in association with resistance to chemotherapeutic drugs (Zhou *et al*, 2012). Although we did not observe such effects in our *in vitro* models, lactate levels were increased in the drug-resistant xenografts and the concomitant upregulation of the glucose transporter GLUT-1 in CAL^R^ relative to CAL^S^ tumours is consistent with a switch to glycolytic metabolism. Levels of GLUT-1 were comparable between CAL^R^ and CAL^S^ in 2D monolayers and 3D spheroids, where lactate levels were unchanged, indicating that the Warburg effect observed in tumours is not an inherent feature of the parental cells rather it is a consequence of the *in vivo* microenvironment. Oxidative stress and hypoxia may be underlying causes of this glycolytic switch as they can induce the expression of hypoxia inducible factor-1 alpha, which can, in turn, upregulate the transcription of GLUT-1 (Verschoor *et al*, 2010; Brahimi-Horn *et al*, 2011). Hypoxia is also a major cause of treatment-resistance in HNSCC patients (Koukourakis *et al*, 2006; Gee *et al*, 2010). Further work is required to characterise the physiologic phenotype of CAL^R^ and CAL^S^ tumours and assess if any differences could account for the increase in GLUT-1 expression and lactate levels observed here.

High levels of cellular bioenergy metabolite levels including the phosphagen metabolite PCr have been reported to be linked to chemoresistance (Kaplan *et al*, 1991; Zhou *et al*, 2012) and could indicate an enhancement in energy metabolism required for sustaining increased cell proliferation (Cairns *et al*, 2011). We also observed increased aspartate and alanine in CAL^R^ relative to CAL^S^ tumours and spheroids. This is of interest as aspartate constitutes a pivotal component of the malate-aspartate shuttle, a process that enables efficient energy production in the cell (Wallace, 2012). A key role has recently emerged for glutamine-derived aspartate in the generation of reducing equivalents necessary for protection against oxidative stress (Son *et al*, 2013). Although such functions would fit with the demands for improved bioenergetics evident in the aggressive growth phenotype of CAL^R^ tumours and spheroids, further work is necessary to assess if the rise in aspartate (and alanine) observed here are linked to this process or other mechanisms.

Our data point to increased GPC as a metabolic biomarker of acquired EGFR TKI resistance in the CAL^R^/CAL^S^ model that is independent of any growth environment or phenotype. This is of interest as choline metabolism is altered in cancer cells and both PC and GPC are increased with malignancy, not only as a result of increased cell proliferation but also because of a direct oncogenic protein regulation (Glunde *et al*, 2011). GPC levels were also increased in the sensitive CAL^S^ but not the resistant CAL^R^ cells following acute exposure to the EGFR TKI gefitinib, indicating that the processes leading to the rise in GPC are triggered by drug treatment and persist when the cells become chronically resistant to drug. An increase in GPC has been observed with various chemotherapeutic and targeted anti-cancer agents (Beloueche-Babari *et al*, 2010), thus it is unlikely to be specific to EGFR TKIs.

The activity of GPC-pd, the enzyme that cleaves GPC into choline and glycerol-3 phosphate, was significantly reduced in CAL^R^ relative to CAL^S^ cells. In the absence of significant differences in fatty acyl signals between the two cell lines, that is, no major changes in lipid breakdown, we conclude that inhibition of GPC-pd is likely to be the major cause for the elevation in GPC content in the drug-resistant CAL^R^ cells. Further work is necessary to establish the molecular and cellular mechanisms linking acquired resistance to EGFR TKIs in this model and the ensuing inhibition of GPC-pd.

Finally, to test if our metabolic signature of resistance is applicable to other HNSCC models, we tested a second sensitive/resistant cell line pair: PJ^S^/PJ^R^. We found that in this case acquired resistance to EGFR TKI was also associated with changes in choline metabolism (indicating altered membrane phospholipid turnover) but, unlike the CAL^R^/CAL^S^ model, a reduction in GPC and PC levels was observed. The basis for this difference is unclear but may be related to differences in underlying drug resistance mechanisms, including polyploidy (previously linked to resistance to cytotoxic drugs (Puig *et al*, 2008; Shen *et al*, 2008)), which was observed in the PJ but not the CAL model. Differences in the anatomical site of origin of the cells (tongue for CAL^S^/CAL^R^ and oral cavity (excluding tongue) for PJ^S^/PJ^R^) may also be involved. Alterations in GPC levels have previously been reported in human cancer cells exhibiting multidrug resistance but the direction of the change was cell line dependent and likely to reflect cellular processes accompanying the induction of the drug resistance phenotype (Kaplan *et al*, 1991), in agreement with our findings. Clearly, analysis of a larger number of isogenic sensitive/resistant cell line pairs is required to assess the general applicability of the metabolic signatures observed here, and unravel the mechanisms underlying them.

A key objective of our study was to identify metabolic characteristics of acquired resistance to EGFR TKIs that could be developed as non-invasive imaging biomarkers. The glycolytic phenotype observed in the tumours can be monitored with ^18^F-fluorodeoxyglucose (FDG)-PET, the gold-standard method for clinical metabolic imaging, as well as lactate-selective *in vivo* ^1^H NMR spectroscopic imaging, which have both been used clinically in HNSCC (Le *et al*, 2008; Jansen *et al*, 2012). The changes in GPC and total choline signal, as well as Cr+PCr observed with acquired resistance can also be monitored with standard *in vivo* ^1^H NMR spectroscopy techniques as shown in previous patient studies (including HNSCC) (King *et al*, 2010; Kim *et al*, 2011; Germuska *et al*, 2012; Jansen *et al*, 2012). *Ex vivo* analysis of tumour tissue may be particularly useful for monitoring metabolites not measurable by currently available imaging techniques, for example, aspartate and alanine as previously documented in clinical HNSCC samples (Somashekar *et al*, 2011). Longitudinal studies assessing the metabolic characteristics of human HNSCC and correlating them with patient outcome are, however, necessary to assess the utility of metabolic biomarkers for patient follow-up.

In summary, this study has revealed metabolic signatures associated with acquired EGFR TKI resistance that may be linked to the underlying drug resistance mechanisms as well as aggressive growth patterns in HNSCC models. The changes reported here can be monitored through *in vivo* as well as *ex vivo* examinations of patient tumours, highlighting their potential for translation to the clinic.

## Figures and Tables

**Table 1:** Metabolite content of CAL^R^ and CAL^S^ HNSCC human tumour xenografts as detected by ^1^H and ^31^P NMR spectroscopy analysis of excised tumour tissue

	**CAL**^**S**^	**CAL**^**R**^	***P***^a^
**^1^H Metabolites (au g^−1^ wet tissue)**			
BCAA	59±6	108±11	**0.007**
Lactate	40±4	67±8	**0.02**
Alanine	36±4	77±9	**0.006**
Acetate	12±2	9±1	0.16
Glutamate	65±6	83±9	0.14
Glutamine	39±4	41±5	0.74
Glutathione	25±4	24±4	0.86
Aspartate	1.8±0.5	2.7±0.2	**0.01**
Cr+PCr	46±3	62±7	0.07
PC	45±5	45±5	0.96
GPC	27±4	58±9	**0.02**
Total choline	75±7	118±16	**0.05**
Myo-inositol	10±1	8±1	0.25
**^31^P Metabolites (*μ*mol g^−1^ wet tissue)**			
PCr	0.36±0.08	0.94±0.16	**0.02**
Pi	2.14±0.44	2.44±0.22	0.57
GPC	0.11±0.03	0.53±0.06	**0.001**
GPE	0.05±0.02	0.19±0.03	**0.009**
PC	0.33±0.03	0.34±0.03	0.84
PE	0.55±0.07	0.31±0.04	**0.02**

a Two-tailed unpaired Student's *t*-test, *n*=5. Bold denotes statistically significant *P* values.

**Table 2:** ^1^H NMR spectroscopy-detectable metabolite levels in CAL^R^ and CAL^S^ cells grown as 2D monolayers or as 3D spheroids

	**CAL**^**S**^	**CAL**^**R**^	***P***^a^
**2D cell metabolites (au per cell volume)**			
BCAA	13±1	15±1	0.16
Lactate	5.20±0.60	5.40±0.40	0.66
Alanine	9.50±1.20	9.20±0.80	0.84
Glutamate	5.50±0.50	6.00±0.50	0.54
Aspartate	0.49±0.06	0.34±0.04	0.08
Cr+PCr	2.20±0.20	1.9±0.20	0.44
PC	10±2	10±1	0.82
GPC	1.90±0.3	3.90±0.6	**0.02**
PE	0.61±0.06	0.54±0.09	0.53
**3D spheroid metabolites (ratio to PC)**			
BCAA	7.30±1.30	4.50±0.60	0.1
Lactate	1.52±0.16	1.34±0.28	0.58
Alanine	1.30±0.30	2.70±0.50	**0.038**
Glutamate	0.54±0.17	1.27±0.55	0.15
Aspartate	0.05±0.03	0.15±0.02	**0.025**
Cr+PCr	0.56±0.07	0.87±0.04	**0.003**
GPC	0.32±0.02	0.63±0.07	**0.007**
PE	0.36±0.10	0.19±0.07	0.29

a Unpaired 2-tailed Student's *t*-test, *n*⩾3. Bold denotes statistically significant *P* values.

**Table 3:** ^1^H NMR spectroscopy-detectable metabolite levels in PJ^R^ and PJ^S^ cells grown as 2D monolayers

**2D cell metabolites (au/cell volume)**	**PJ**^**S**^	**PJ**^**R**^	***P***^a^
BCAA	4.80±0.25	5.4±0.35	0.23
Lactate	6.90±0.80	7.50±1.00	0.66
Alanine	2.20±0.30	2.20±0.38	0.96
Glutamate	2.10±0.20	2.40±0.30	0.46
PC	17±2	11±1	**0.05**
GPC	1.50±0.10	0.54±0.03	**0.01**
PE	0.50±0.14	0.20±0.05	0.17

a Unpaired 2-tailed Student's *t*-test, *n*=3. Bold denotes statistically significant *P* values.
