# Supplementary material for: Acquired resistance to EGFR tyrosine kinase inhibitors alters the metabolism of human head and neck squamous carcinoma cells and xenograft tumours
Source: Br J Cancer. 2015 Mar 5;112(7):1206–14. doi: 10.1038/bjc.2015.86 (PMC4385966; doi:10.1038/bjc.2015.86)
Supplement: Supplementary Table S1 [file bjc201586x1.doc]

**Supplementary Table S1:** Changes in levels of fatty acyl chains in CAL^R^ relative to CAL^S^ cells as detected by ^1^H NMR spectroscopy of the organic phase of cell extracts.

| **Lipid signal peaks** | **% change in CAL^R^ relative to CAL^S^** | ***P^#^*** |
| --- | --- | --- |
| 0.9 ppm (CH_3_-) | 106±5 | 0.12 |
| 1.3 ppm (-CH_2_)_n_ -) | 108±3 | 0.34 |
| 1.6 ppm (-OOCCH_2_-CH_2_ -) | 115±6 | 0.12 |
| 2.0 ppm (-CH=CHCH_2_ -) | 100±3 | 0.99 |
| 2.3 ppm (-OOCCH_2_ -) | 101±3 | 0.81 |
| 2.8 ppm (=CH-CH_2_-CH=) | 98±1 | 0.90 |
| 5.3 ppm (-CH=CH-) | 90±2 | 0.06 |

Data are expressed as ratios of peaks to phosphatidylcholine within each sample.

^#^: unpaired 2-tailed Student’s t-test, n=3.
