# Supplementary figures and images for: Acquired resistance to EGFR tyrosine kinase inhibitors alters the metabolism of human head and neck squamous carcinoma cells and xenograft tumours
Source: Br J Cancer. 2015 Mar 5;112(7):1206–14. doi: 10.1038/bjc.2015.86 (PMC4385966; doi:10.1038/bjc.2015.86)

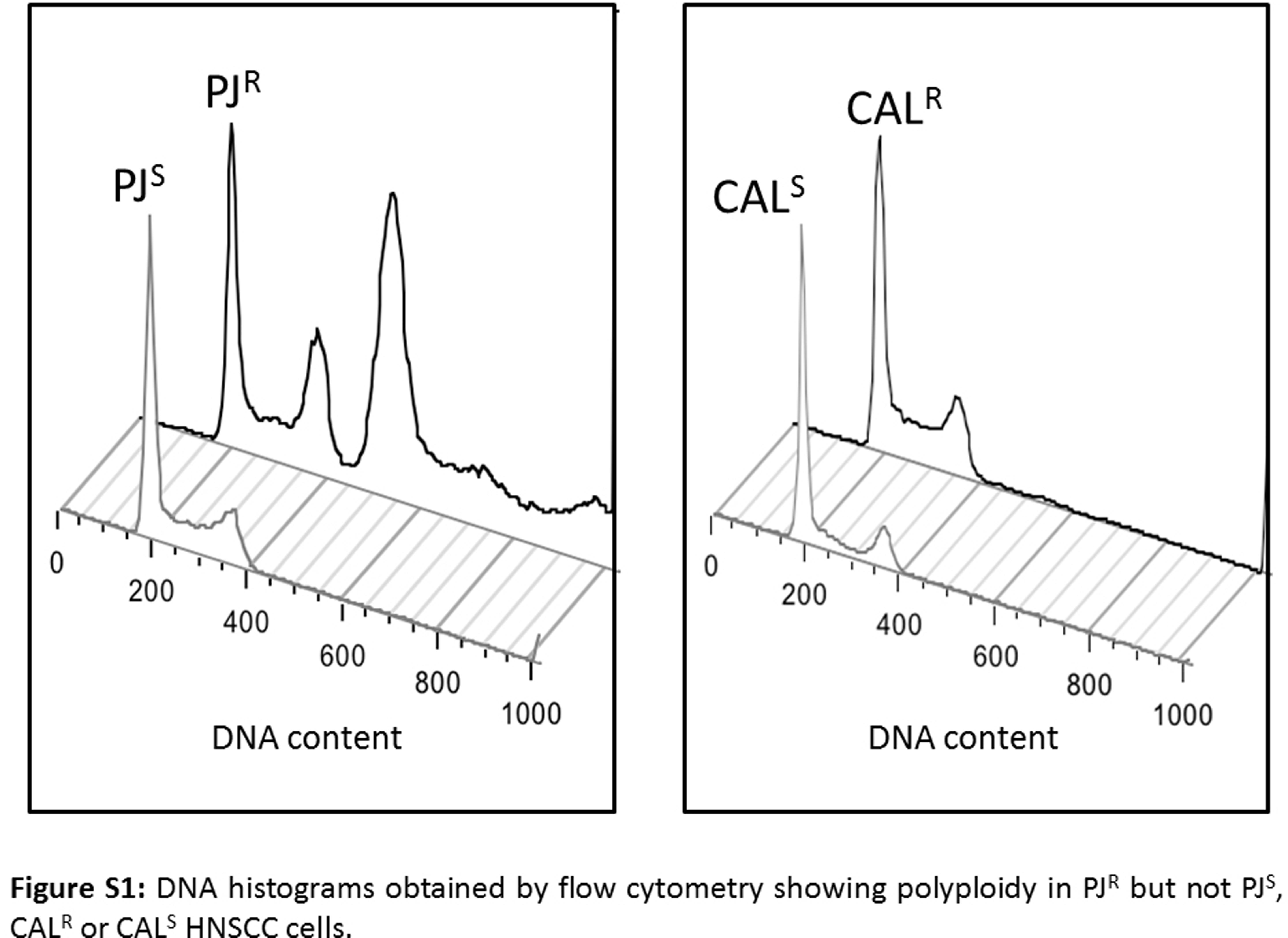

Supplement: Supplementary Figure S1 [file bjc201586x2.tif]

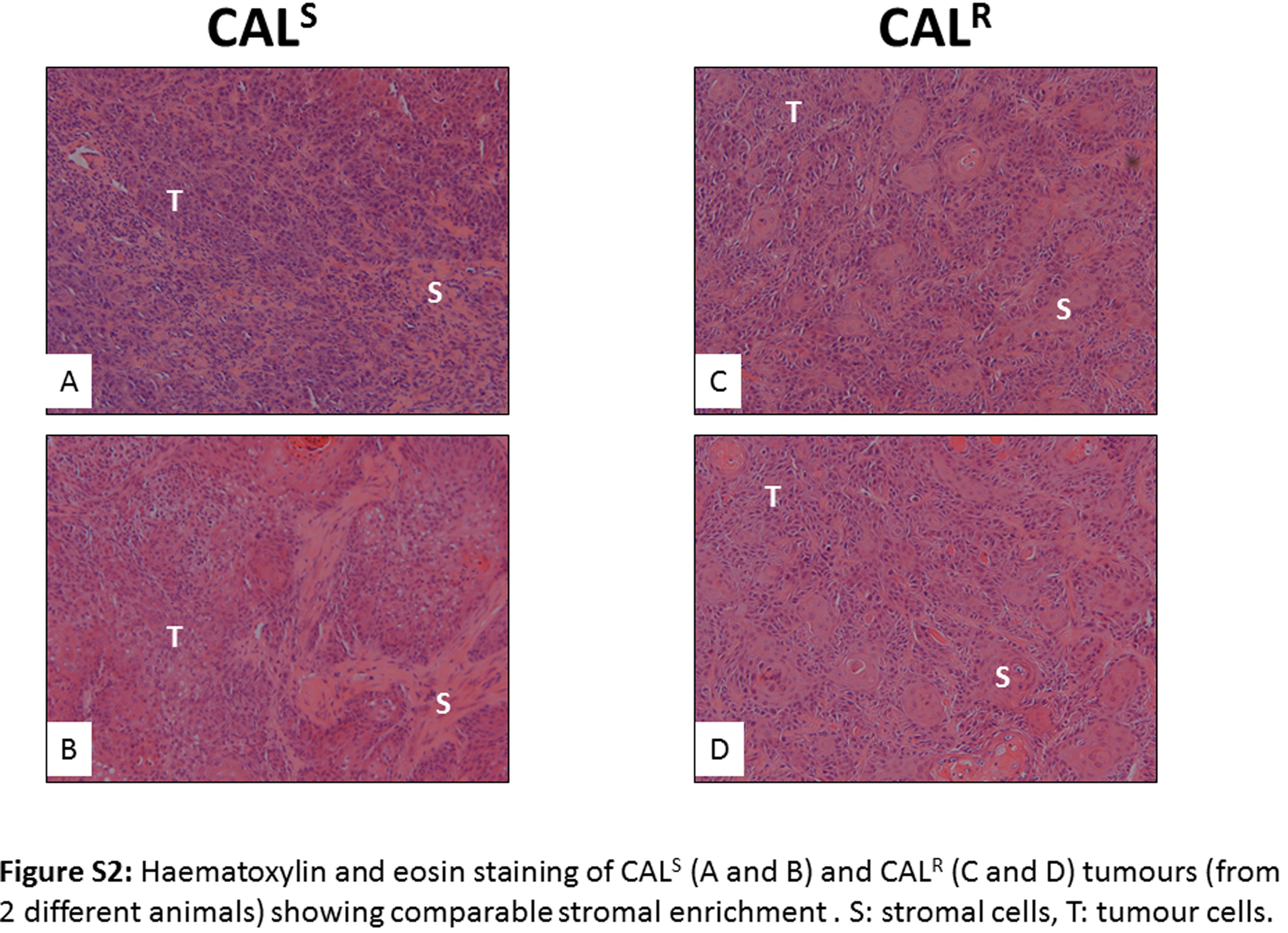

Supplement: Supplementary Figure S2 [file bjc201586x3.tif]
